# Leaf Traits Mediate Phyllosphere Bacterial Community Assembly and Their Role in Degrading Traffic-Derived Polycyclic Aromatic Hydrocarbons

**DOI:** 10.3390/microorganisms14020334

**Published:** 2026-02-01

**Authors:** Zheng Yang, Qingyang Liu, Shili Tian, Yanju Liu, Ming Yang, Ying Liang, Xin Chen

**Affiliations:** 1 Beijing Milu Ecological Research Center, Beijing 100076, China; yz@milupark.org.cn (Z.Y.); lvrichu@126.com (X.C.); 2 College of Ecology and the Environment, Nanjing Forestry University, Nanjing 210037, China; 3 Institute of Analysis and Testing, Beijing Center for Physical and Chemical Analysis, Beijing Academy of Science and Technology, Beijing 100089, China

**Keywords:** phyllosphere, polycyclic aromatic hydrocarbons (PAHs), phytoremediation, traffic pollution, microbiota

## Abstract

Transport emissions are a major source of urban polycyclic aromatic hydrocarbons (PAHs), posing risks to human health. While plant leaves and their epiphytic microbes contribute to PAH degradation, how plant traits and environmental factors affect this process remains unclear. This study examined 20 tree species in Beijing’s traffic corridors to explore PAH enrichment on leaves and the structure of phyllospheric bacterial communities. Results show that leaf area, morphology, and sampling height significantly influenced bacterial community assembly. Normalized Stochasticity Ratio (NST) analysis indicated that deterministic processes dominate on medium-sized leaves (11.8–40.1 cm^2^), simple leaves, and those below 2.3 m or above 3 m in height, whereas stochastic factors prevail on nano leaves, compound leaves, and leaves at low-position (<2.3 m). Although low-molecular-weight PAHs (2–4 rings) were predominant in leaves, Mantel tests revealed significant positive correlations between bacterial communities and high molecular weight PAHs (4–6 rings), such as benz(a)anthracene, benzo[e]pyrene, and picene. Spearman analysis identified 10 dominant bacterial taxa with PAH degradation potential, including *Kocuria rosea*, *Serratia symbiotica*, *Massilia* sp. WG5, and seven unclassified species from *Hymenobacter*, *Sphingomonas*, *Roseomonas*, *Curtobacterium*, and *Deinococcus*. Functional Annotation of Prokaryotic Taxa(FAPROTAX) prediction further associated 14 species across six genera, including *Acinetobacter*, *Nocardioides*, *Gordonia*, *Rhodococcus*, *Clostridium_sensu_stricto*_18, and *Geobacter*, with PAH degradation function. This work clarifies the composition and function of phyllospheric PAH-degrading bacteria in an urban traffic environment, offering a theoretical basis for enhancing degradation via bacterial consortia, biosurfactants, and optimized plant selection.

## 1. Introduction

Transport emissions are a principal source of PAHs in urban air [1], notably particulate-phase PAHs [2]. Direct exposure to PAHs can elicit various toxic effects in humans [3], and the elevated concentrations of PAHs near traffic sources, in particular, constitute a substantial risk to human health [4]. In the urban environment, the natural degradation of PAHs is considerably hindered owing to their chemical stability and hydrophobicity [1,2,3]. The 31 target PAHs analyzed in this study encompassed not only the 16 USEPA priority PAHs but also additional compounds, including retene, cyclopenta[cd]pyrene, and picene [3]. These were selected based on (1) their documented prevalence in urban traffic emissions and traffic airborne particulate matter in China [3], (2) emerging toxicological data indicating significant mutagenic and carcinogenic potential comparable to or exceeding some priority PAHs [1,2,3], and (3) inclusion in broader environmental monitoring frameworks, aligning with the need to assess a more comprehensive profile of traffic-derived PAH pollution [3].

Although photodegradation is an effective process for PAH degradation, in urban settings, its efficiency is limited by environmental constraints [5,6]. Phytoremediation is a bioremediation technique that utilizes plants and their associated microorganisms to remove various environmental contaminants [7]. For environmental PAH contamination, phytoremediation holds significant potential [7,8]. There are multiple pathways for the phytoremediation of PAHs, among which plants themselves possess the capacity for uptake and accumulation [9,10]. However, plants typically lack complete PAH degradation pathways [11]. They can only rely on their inherent enzyme systems or employ transgenic approaches to introduce or enhance the plant’s capacity to degrade PAHs [11,12]. The rhizospheric degradation pathway facilitates the biodegradation of PAHs through root exudates [13] and is primarily targeted at soil contamination. Consequently, the aforementioned pathway is not suited to the context of phytoremediation in urban traffic corridors. Plant–microbe synergistic systems represent an effective method for removing urban air pollutants and can be applied to remediate environmental PAHs [9,14]. The symbiotic relationship between plants and specific microorganisms can significantly enhance the degradation efficiency of PAHs [15,16], in some cases approaching complete degradation [16,17]. However, the efficiency of PAH degradation by phyllospheric microorganisms remains highly variable and is likely influenced by host plant characteristics. Research indicates that the composition and function of these phyllospheric communities vary significantly with plant species and their distinct leaf surface environments (e.g., leaf morphology, area, and sampling height) [13,18]. Nevertheless, a systematic understanding of how these leaf traits and environmental factors drive the assembly of phyllospheric bacterial communities and, consequently, their PAH degradation potential, particularly for recalcitrant high molecular weight (HMW) PAHs, is still lacking.

Plant foliage serves as a primary pathway for the interception of airborne PAHs and has been demonstrated to remediate PAHs in urban environments [19,20]. Microorganisms residing on leaf surfaces can effectively degrade intercepted particulate-phase PAHs [21]. However, the efficiency of PAH degradation by phyllospheric microorganisms remains variable. Research indicates that low-molecular-weight (LMW) PAHs are more readily degraded by microbes compared to HMW PAHs, while the degradation of HMW PAHs requires the involvement of specific microbial communities or enzymatic processes [22]. Plant foliage harbors naturally occurring microbial communities responsible for pollutant degradation. The composition and function of these communities vary with plant species and season [13,18,23]. Consequently, the structure of the phyllospheric microbial community differs across various plant species and their distinct leaf surface environments. Furthermore, the microbial species and their capacity to degrade various PAHs on plant leaves remain largely uncharacterized. Consequently, a targeted approach for enhancing the plant–microbe synergistic degradation of specific PAHs can be designed based on the specific contamination profile, plant species, and its inherent phyllospheric bacterial community.

This study focuses on 20 common species of roadside arboreal plants in Beijing’s traffic corridors. By analyzing the concentration of PAHs enriched on their leaf surfaces and employing 16S rRNA sequencing to characterize the phyllospheric bacterial communities, it aims to identify bacterial taxa with the capability or potential to degrade these PAHs. The influence of leaf morphology and spatial factors on these communities will also be assessed. Therefore, this study hypothesizes that the assembly of phyllospheric bacterial communities on roadside trees is deterministically driven by specific leaf traits (e.g., area, morphology) and spatial factors (e.g., height), and that these shaped communities, in turn, possess distinct potentials for degrading PAHs, especially the high molecular weight compounds prevalent in traffic emissions. To test this hypothesis, we conducted a field study on 20 common tree species in Beijing’s traffic corridors. By analyzing the enrichment of PAHs on leaf surfaces and characterizing the phyllospheric bacterial communities via 16S rRNA sequencing, we aim to (1) identify the key leaf traits and environmental factors governing bacterial community assembly, (2) elucidate the correlations between bacterial community structure and specific PAH compounds, and (3) pinpoint the bacterial taxa with demonstrated potential for PAH degradation. Our work provides a novel, trait-based perspective on the plant–microbe interactions in urban air phytoremediation and offers a theoretical foundation for enhancing PAH degradation through optimized plant selection and targeted microbial consortia.

## 2. Methods

### 2.1. Sample Collection and Pre-Processing

A total of 20 plant species were collected from the traffic corridors along Beijing’s West Third Ring Road (116°18′14″ N, 39°54′2″ E) in July 2022 for phyllosphere microbial sequencing and PAH analysis. The specific plant species are listed in Table 1. Three mature and healthy trees of similar sizes and ages were selected for each species. Intact leaves of similar ages (approximately 1 year old) were collected from the direction of the highways. Approximately 100 leaves were collected from three parallel trees per species [19]. After collection, the leaves were kept in polyethylene bags and transported to the laboratory at 4 °C.

### 2.2. PAH Analysis in Leaf Samples

The analysis of phyllospheric PAHs followed our established method [19]. Briefly, approximately 200 g of leaf samples were crushed and homogenized. Subsequently, 5 g of the resulting powder was subjected to ultrasonic extraction using a mixture of n-hexane and dichloromethane (1:1, *v*/*v*). Detailed information is provided in the Supplementary Materials (Text S1). The extract was purified via a solid-phase extraction column (silica gel and neutral alumina) and analyzed by gas chromatography–mass spectrometry (GC–MS, QP2010Ultra Shimadzu, Kyoto, Japan) equipped with a DB-EUPAH capillary column (30 m × 0.25 mm, 0.25 μm). The method demonstrated good linearity (R^2^ > 0.99) from 1 to 500 ng mL^−1^, with limits of quantification for individual PAHs ranging from 0.8 to 2.8 ng g^−1^ and recovery rates of 71–98%.

### 2.3. Phyllosphere Microbiome Sampling

During sample pre-processing for sequencing, leaves were divided into two groups: the leaf surface group (Surface) and the endophytic control group (Surface CK). For the leaf surface group, sterile ultra-pure water and sterile pipette tips were used to spray-wash both sides of each leaf five times. The leaves were then successively fully immersed and washed three times in three separate vessels, each containing 100 mL of sterile ultra-pure water. All spray-wash and immersion-wash solutions were collected and filtered using a sterilized stainless-steel positive pressure filtration unit fitted with MCE membranes (Millipore, GSWP14250,Burlington, MA, USA). The filter membranes were preserved on dry ice for subsequent sequencing. For the endophytic group, leaves underwent the identical washing procedure as the surface group. After washing, 2 g of leaf tissue was placed into a pre-sterilized 10 mL centrifuge tube containing stainless-steel grinding beads, homogenized for 10 min using a high-throughput tissue homogeniser, and subsequently preserved on dry ice for sequencing. The washing and filtration protocol was adapted from methods commonly used for phyllosphere microbiome studies [13,18].

### 2.4. Plant Trait Measurement and Grouping

The mean leaf area for each plant species sampled and the height of the sampling location above ground level are presented in Table 1. K-means clustering analysis was performed on both the mean leaf area and the sampling height. Each parameter was grouped into four clusters, as indicated by the plateau in variance reduction observed during clustering analysis. The mean leaf area groups (Group-Area) are designated as follows: Macro leaf (A1), Micro leaf (A2), Meso leaf (A3), and Nano leaf (A4). The sampling height groups (Group-Height) are designated as Low-level (H1), Ground-level (H3), Mid-height (H4), and Elevated (H2). The corresponding threshold ranges for leaf area were A1 (>40.1 cm^2^), A2 (20.1–40.1 cm^2^), A3 (11.8–20.1 cm^2^), A4 (<11.8 cm^2^); and for sampling height: H1 (1.9–2.3 m), H3 (0–1.7 m), H4 (3.0–3.7 m), H2 (4.4–5.5 m). Plants were also categorized by leaf type: Single leaf plants (SL) and Compound leaf plants (CL). Detailed grouping information is provided in Table 1.

### 2.5. Data Analysis

Bioinformatics and statistical analyses were primarily conducted on the Majorbio Cloud Platform and in R (version 4.3.1; R Foundation for Statistical Computing). K-means clustering was used to categorize plants by leaf area and sampling height. A suite of bioinformatic and statistical analyses were conducted to examine the bacterial communities [13,18]. Sequencing depth was evaluated using rarefaction curves. Core microbiome analysis defined taxa consistently present (≥80% prevalence) within each group, while Venn diagrams visualized overlaps between the Surface and endophytic (Surface CK) communities. The relative contributions of deterministic versus stochastic assembly processes were quantified via the NST analysis. Furthermore, Mantel tests assessed correlations between overall community structure (Bray–Curtis distances) and PAH concentration profiles, considering both the complete dataset and subgroups based on leaf traits [13,22]. Spearman correlation was used to test relationships between (1) the top 20 dominant bacterial species and PAH concentrations, and (2) taxa predicted to be involved in aromatic compound degradation of PAHs. The FAPROTAX database was used for functional prediction, focusing on the aromatic compound degradation group. Variance Inflation Factor (VIF) analysis was applied to screen PAH environmental factors. OTUs were clustered using Uparse and annotated against the Greengenes database. Detailed protocols for PAH analysis and extended data analysis descriptions are provided in the Supplementary Materials (Texts S1 and S2).

## 3. Results

### 3.1. Bacterial Community Composition

**Figure 1:**
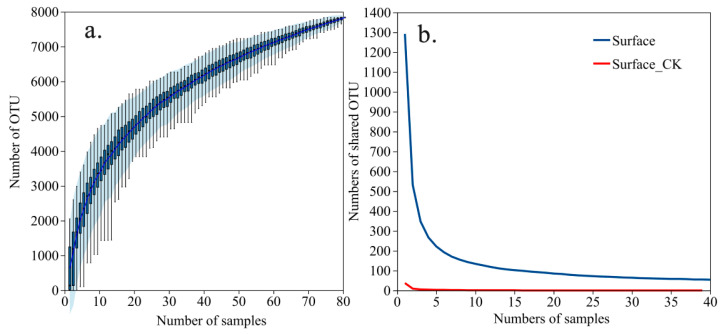
Sequencing depth and core microbiome analysis. (**a**) Rarefaction curves show sufficient sequencing depth to capture bacterial diversity. (**b**) Core OTU analysis reveals a more homogeneous endophytic community (Surface CK) compared to the diverse and variable leaf surface community (Surface).

**Figure 2:**
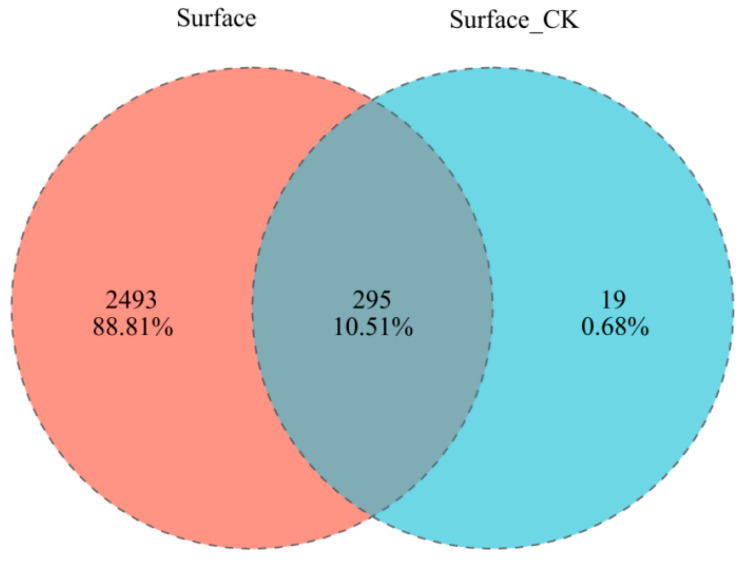
Phyllosphere bacterial community is highly distinct from endophytes. Venn diagram illustrates the limited sharing of bacterial species (295 species) between the leaf surface and endophytic compartments, with the phyllosphere harboring the vast majority of unique species (2493).

The concentrations of PAHs detected on the leaves of the 20 tree species have been published previously [19]. The total PAH concentrations ranged from 12.4 to 68.4 ng g^−1^, with low-molecular-weight PAHs (2–4 rings) being the most frequently detected and abundant. Sequencing yielded 7836 OTUs from the phyllosphere, with rarefaction curves confirming sufficient depth (Figure 1a). Core microbiome and Venn diagram analyses revealed a distinct and highly diverse leaf surface community (Surface), harboring 2493 unique species, compared to a more homogeneous endophytic community (Surface CK) with only 19 unique species (Figure 1b and Figure 2). As the Surface group represented the vast majority (99.32%) of the detected bacterial diversity, it was used for all subsequent analyses.

### 3.2. Leaf PAH Profile and Valid Environmental Factors

**Figure 3:**
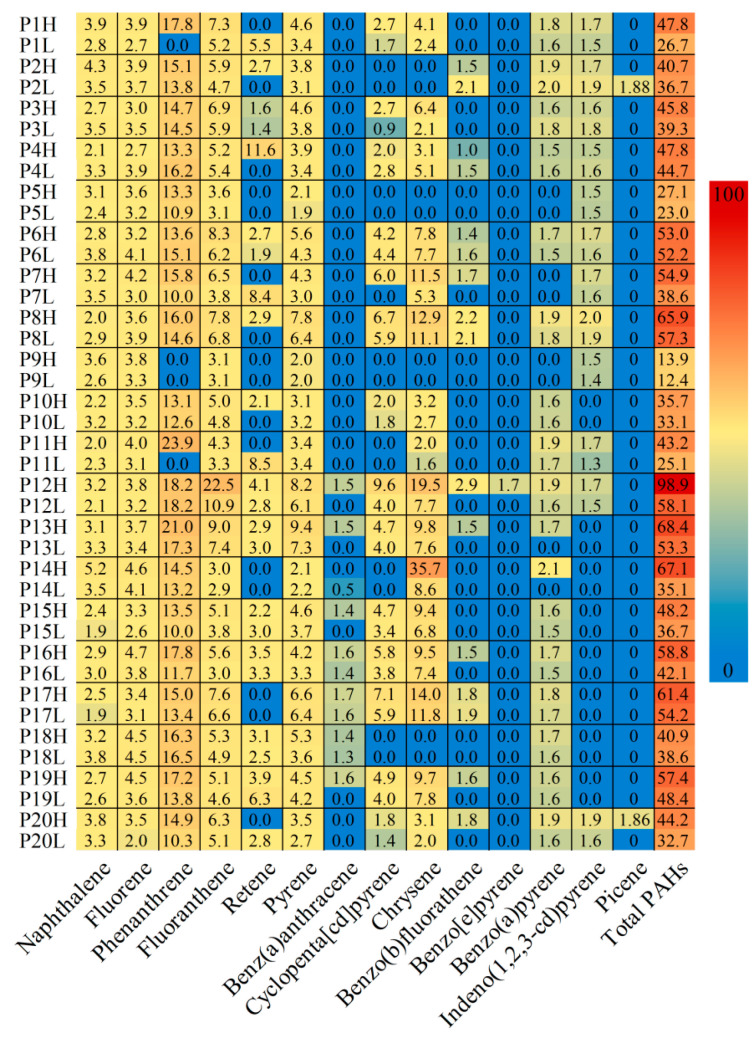
Composition and concentration profile of polycyclic aromatic hydrocarbons (PAHs) detected on leaf surfaces. Heatmap displaying the concentrations of the 14 detected PAHs across all leaf samples from the 20 tree species (P1–P20). The PAHs are ordered by molecular weight, revealing that lower molecular weight (2–4 ring) PAHs, such as naphthalene, fluorene, and phenanthrene, were the most frequently detected and abundant contaminants intercepted by leaves in the traffic corridors. Sample labels P1 through P20 correspond to the 20 plant species listed in Table 1. For certain species, the suffixes Hand L denote independent replicate samples, with H representing high and L representing low, respectively.

Analysis of leaf samples revealed 14 out of 31 target PAHs, predominantly LMW (2–4 ring) compounds (Figure 3), indicating that leaves primarily intercept LMW PAHs in Beijing’s traffic corridors.VIF analysis revealed that fluoranthene exhibited severe multicollinearity (VIF > 10). It was therefore excluded, which resulted in 13 PAHs being retained as valid environmental factors for the ensuing analysis.

**Figure 4:**
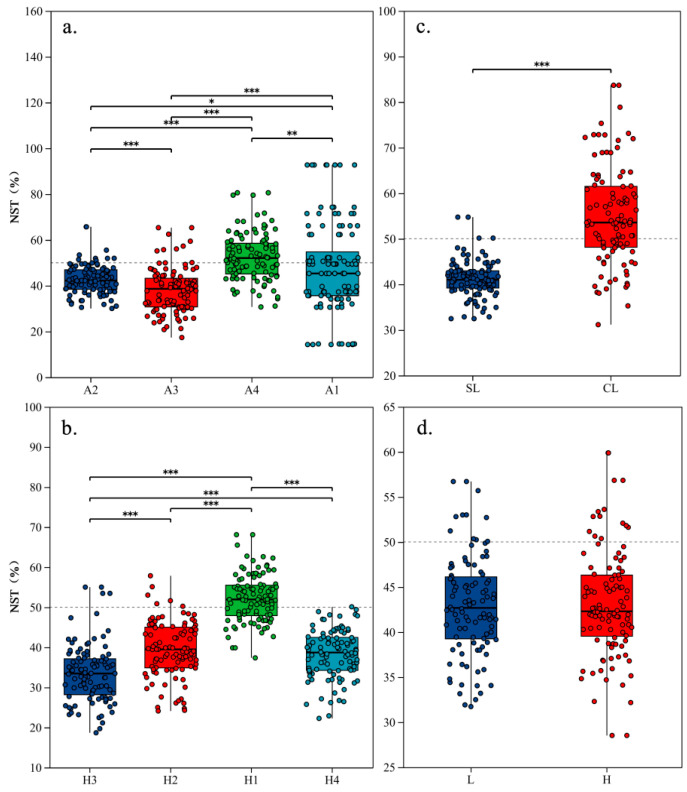
Deterministic and stochastic processes in phyllosphere bacterial community assembly shaped by leaf traits and environment. Normalized Stochasticity Ratio (NST) analysis quantifying the influence of deterministic (<50%, environmental selection) versus stochastic (>50%, neutral processes) assembly on bacterial communities. (**a**) leaf area, (**b**) sampling height, (**c**) leaf morphology, and (**d**) leaf surface PAH concentration. Deterministic processes dominated on micro (A2) and meso (A3) leaves, at ground (H3), mid (H4), and elevated (H2) heights, and on single leaves (SL). Stochasticity prevailed on nano (A4) and macro (A1) leaves, at low heights (H1), and on compound leaves (CL). The letters L and H in panel (**d**) denote low and high PAH concentration levels, respectively. PAH concentration had no significant effect. Significance levels: * 0.01 < *p* ≤ 0.05, ** 0.001 < *p* ≤ 0.01, *** *p* ≤ 0.001.

NST analysis revealed that bacterial community assembly was primarily governed by deterministic processes (environmental selection) on micro (A2) and meso (A3) leaves, at ground (H3), mid (H4), and elevated (H2) heights, and on single leaves (SL). In contrast, stochastic processes dominated on nano (A4) and macro (A1) leaves, at low heights (H1), and on compound leaves (CL) (Figure 4). Notably, PAH concentration level (categorized as high or low) had no significant effect on community assembly. In summary, deterministic assembly was strongest for medium-sized (11.8–40.1 cm^2^), single leaves positioned below 2.3 m or above 3 m in height.

### 3.3. Bacterial Associations with PAHs

**Figure 5:**
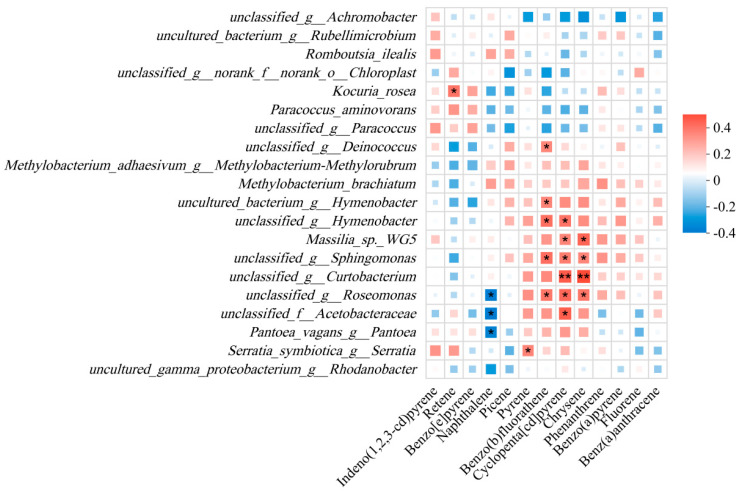
Correlations between dominant phyllosphere bacteria and specific PAH compounds. Spearman correlation heatmap between the relative abundance of the top 20 dominant bacterial species (genus-level) and the concentrations of valid PAH environmental factors. Square size and color intensity represent the correlation coefficient (R value). Key positive correlations (red) identified potential PAH-degrading taxa, such as *Kocuria rosea* with retene, *Serratia symbiotica* with pyrene, and unclassified *Hymenobacter* and *Sphingomonas* species with high molecular weight PAHs like benzo(b)fluoranthene and cyclopenta[cd]pyrene. Significance level: * 0.01 < *p* ≤ 0.05; ** 0.001 < *p* ≤ 0.01. Note: Fluoranthene (Flt) was excluded from this correlation analysis due to multicollinearity, resulting in 13 PAHs being analyzed.

Correlation analyses revealed significant associations between bacterial communities and HMW PAHs, dependent on leaf traits. Spearman analysis identified 10 dominant bacterial species showing significant correlations with specific PAHs (Figure 5). Notably, *Kocuria rosea*, *Serratia symbiotica*, and *Massilia* sp. WG5, along with multiple unclassified species from genera like *Hymenobacter* and *Sphingomonas*, were positively correlated with various PAHs, suggesting their degradation potential.

**Figure 6:**
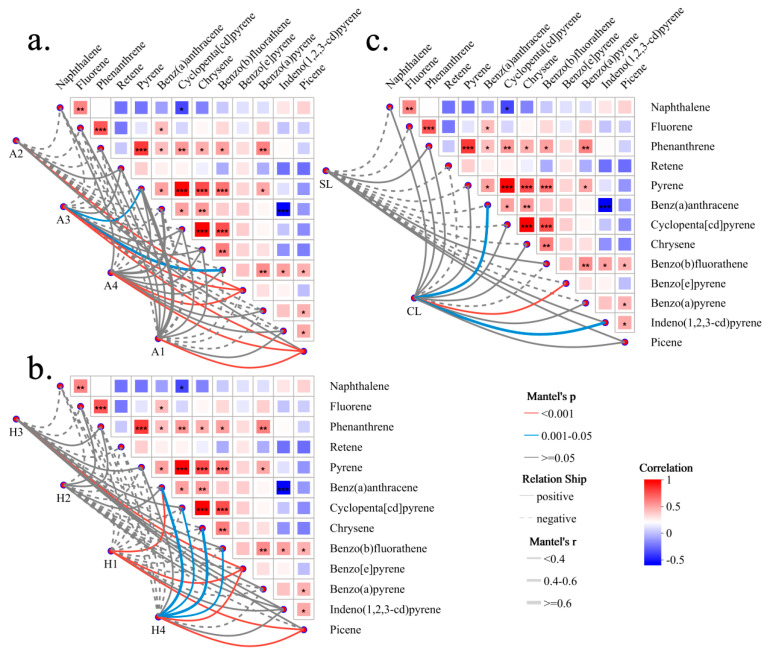
Links between overall bacterial community structure and high molecular weight PAHs are dependent on leaf traits. Mantel test results examining the correlation between the overall bacterial community structure (Bray–Curtis distance) and PAH profiles, stratified by (**a**) leaf area, (**b**) sampling height, and (**c**) leaf morphology. Line thickness indicates the Mantel’s r value. The analysis reveals that bacterial communities on meso leaves (A3), at mid–low heights (H1, H4), and on compound leaves (CL) are significantly associated with 4–6 ring, high molecular weight PAHs (e.g., benz(a)anthracene, benzo[e]pyrene, picene). No significant correlations were found with low-molecular-weight PAHs. Significance levels: * 0.01 < *p* ≤ 0.05, ** 0.001 < *p* ≤ 0.01, *** *p* ≤ 0.001. Note: The PAH distance matrix used in the Mantel test excluded fluoranthene due to multicollinearity (VIF > 10).

Mantel tests further demonstrated that the overall bacterial community structure was significantly correlated with HMW PAHs (4–6 rings), but not with LMW compounds (Figure 6). Leaf traits exerted a pronounced influence on these community–PAH associations. The strongest links were predominantly observed in three specific contexts: mesophyll leaves (A3), sampling heights classified as mid–low (H1, H4), and compound leaves (CL). Key HMW PAHs involved in these correlations included benz(a)anthracene, benzo[e]pyrene, and picene.

### 3.4. Core PAH-Degrading Taxa Identified

**Figure 7:**
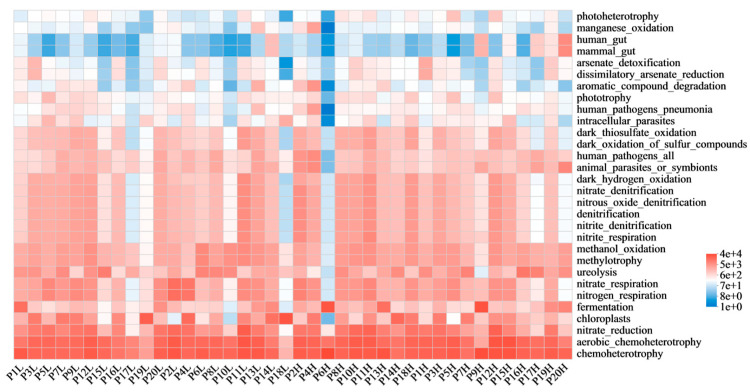
Predicted metabolic functional potential of phyllosphere bacterial communities. Heatmap of the relative abundance of the top 30 predicted functional groups (via FAPROTAX) across all samples. Functions like chemoheterotrophy and aerobic chemoheterotrophy were most abundant. The function aromatic compound degradation showed considerable variation among plant species, with samples like P4H and P13L exhibiting the highest relative abundance, indicating a high potential for microbial bioremediation on specific tree species.

FAPROTAX functional prediction identified the aromatic compound degradation function across the phyllosphere communities, with its relative abundance varying considerably among plant species (Figure 7). The ten samples with the highest relative abundance for this function were P4H (29%), P13L (14.2%), P2H (7.7%), P4L (5.7%), P13H (5.1%), P12H (3.3%), P7L (3.2%), P12L (2.7%), P9L (2.5%), and P2L (2.4%).

**Figure 8:**
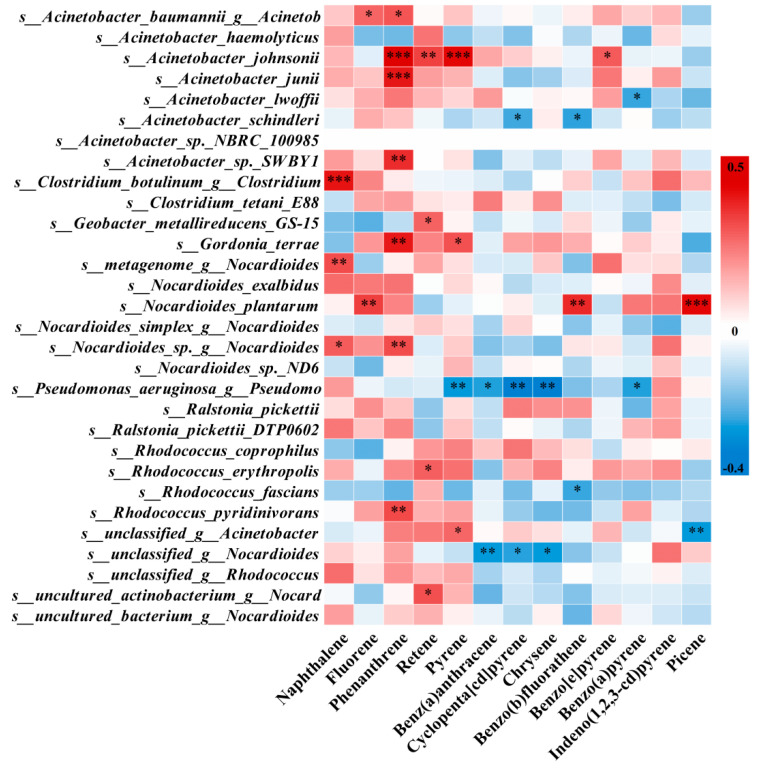
Potential degraders of specific PAHs within the phyllosphere aromatic compound degradation guild. Spearman correlation heatmap between the relative abundance of bacterial species predicted to possess the aromatic compound degradation function and the concentrations of PAHs. This targeted analysis identifies key taxa with confirmed potential for degrading specific PAHs, such as *Acinetobacter johnsonii* for phenanthrene and pyrene, as well as *Nocardioides plantarum* for picene. Positive correlations (red) and negative correlations (blue) suggest the potential roles of these species in the degradation or response to the corresponding PAH within the leaf surface microenvironment. Significance levels: * 0.01 < *p* ≤ 0.05, ** 0.001 < *p* ≤ 0.01, *** *p* ≤ 0.001. Note: Correlation analysis was performed with 13 valid PAH environmental factors, as fluoranthene was excluded due to multicollinearity.

Subsequent correlation analysis linked this functional guild to specific PAHs, pinpointing key taxa with degradation potential (Figure 8). Notably, *Acinetobacter johnsonii* was positively correlated with phenanthrene and benz(a)anthracene, while *Nocardioides plantarum* was associated with picene. In total, 14 species across six genera, including *Acinetobacter*, *Nocardioides*, *Gordonia*, *Rhodococcus*, *Clostridium_sensu_stricto*_18, and *Geobacter*, showed significant positive correlations between their predicted aromatic degradation potential and PAH concentrations.

## 4. Discussion

### 4.1. Phyllosphere Determinants and Core Degraders

Our NST analysis revealed that deterministic processes, driven by environmental PAHs, dominated bacterial community assembly on medium-sized leaves (11.8–40.1 cm^2^), single leaves, and leaves below 2.3 m or above 3 m in height. This likely reflects the dispersion of traffic-derived, particulate-phase PAHs [24] and their differential deposition at various heights [25], interacting with leaf area-dependent accumulation capacity [26]. Mantel tests further demonstrated that bacterial communities on mesophyll leaves (30.1–40.2 cm^2^) at mid–low heights (1.9–3.7 m) and on compound leaves were significantly correlated with multiple PAHs. Notably, all significant correlations involved 4–6 ring, HMW PAHs (e.g., benz(a)anthracene, benzo[e]pyrene, picene). This aligns with traffic emissions being rich in HMW, particulate-phase PAHs [27,28,29], which are more effectively intercepted by lower canopy levels [30,31], explaining the lack of significant correlations at heights >4.4 m.

Leaf traits and environmental factors significantly influence PAH interception and phyllosphere microbial assembly [30,31]. We identified 10 dominant bacterial species positively correlated with specific PAHs, indicating their degradation potential. Three taxonomically defined species exhibited significant positive correlations with specific PAHs, indicating their degradation potential. *Kocuria rosea*, which has been previously documented to degrade naphthalene and phenanthrene [32,33], was correlated with retene. *Serratia symbiotica*, while not directly reported for PAH degradation, belongs to a genus containing strains with aromatic hydrocarbon metabolic potential [34] and was associated with pyrene. Similarly, *Massilia* sp. WG5, a known phenanthrene degrader, isolated from contaminated soil [35], showed correlations with the higher-molecular-weight PAHs, including cyclopenta[cd]pyrene and chrysene.

Seven additional, currently unclassified bacterial species, identified at the genus level, also showed significant positive correlations with PAHs. These genera possess documented environmental resilience or pollutant-degrading capabilities, suggesting a potential role in the phyllosphere PAH degradation process. Specifically, unclassified species of the genus *Hymenobacter*, which were correlated with benzo(b)fluoranthene and cyclopenta[cd]pyrene, are known for their adaptability to extreme environments and their capacity for pollutant degradation [36,37]. An unclassified *Sphingomonas* sp., which was correlated with benzo(b)fluoranthene, cyclopenta[cd]pyrene, and chrysene, belongs to a genus that contains confirmed PAH degraders [38,39]. Similarly, unclassified *Roseomonas* sp., which has been found in hydrocarbon-contaminated soils [40], and unclassified *Curtobacterium* sp., which was correlated with cyclopenta[cd]pyrene and chrysene and is noted for its crucial role in litter decomposition [41], may adapt to or participate in PAH degradation. An unclassified *Deinococcus* sp., which was correlated with benzo(b)fluoranthene, is renowned for both radiation resistance and PAH degradation capabilities [42,43]. Finally, an unclassified species from the family *Acetobacteraceae*, correlated with cyclopenta[cd]pyrene, has been positively associated with PAH levels in sediments [44], indicating a potential ecological role in PAH-contaminated microenvironments.

FAPROTAX and correlation analysis jointly identified 14 species across six genera, whose aromatic compound degradation potential was positively correlated with PAHs. Among them, eight species from the genus *Acinetobacter* can utilize various PAHs as carbon sources [45,46,47], while their degradation efficiency may be inhibited by high substrate concentrations [48] or enhanced by biosurfactants [49]. The eight identified *Nocardioides* species are key PAH degraders that employ enzymes such as ring-hydroxylating dioxygenases [50,51]. Their degradation efficacy is further enhanced through synergistic interactions with other genera, including *Rhodococcus* and *Sphingomonas* [52,53]. Co-metabolism and biosurfactants can mitigate substrate inhibition for this genus [54,55]. Two species of *Gordonia* showed correlations with phenanthrene and pyrene, in line with their documented PAH degradation capabilities [56,57], while their efficiency can be improved via consortia or co-metabolism [58,59]. The single identified *Rhodococcus* species possesses a broad capacity for PAH degradation, transforming these compounds into intermediates [60,61]. However, it is noted that some of these intermediates can exhibit toxicity [62]. Furthermore, one species from each of the genera *Clostridium_sensu_stricto*_18 and *Geobacter* was positively correlated with naphthalene and retene, respectively. Their known anaerobic degradation capabilities [60,63] suggest activity in micro-anaerobic niches within the leaf cuticular wax and potential for synergistic degradation with other anaerobes [64].

### 4.2. Broader Implications and Future Perspectives

Our findings reveal that leaf traits serve as a selective filter for specific phyllospheric bacteria with PAH-degrading potential, yielding insights with broader ecological and practical implications [13,18]. First, beyond their direct role in pollutant breakdown, these epiphytic microbes may enhance plant fitness in polluted environments by alleviating phytotoxic PAH burdens, effectively acting as a frontline defensive symbiont [9,15,17]. Second, while this study centered on surface communities, endophytic bacteria are recognized for their capacity to transform and potentially volatilize PAHs internally, suggesting a complementary degradation route that merits dedicated exploration [11,12,21].

A clear distinction emerged in the fate of different PAHs. Leaves primarily accumulated LMW PAHs, likely due to their higher volatility and gaseous-phase abundance promoting atmospheric deposition [19,20,26].Conversely, the phyllosphere bacterial community structure showed a strong and specific correlation with HMW, particulate-bound PAHs [22,24,27]. This points to a targeted microbial ecological response to these more persistent and typically more toxic compounds [22,27,29]. To fully unravel this process, future research must move beyond correlation by employing techniques like metatranscriptomics or stable isotope probing. These approaches are essential to identify the active microbial metabolic pathways, track the formation and fate of degradation intermediates, and critically assess the environmental risk posed by potentially toxic metabolites generated before complete mineralization is achieved [14].

The enrichment of HMW-PAH specialist communities within the phyllosphere mirrors findings from contaminated soil environments, where degraders of more recalcitrant compounds also become dominant [16,22,50]. This parallel underscores the remarkable adaptive capacity of plant-associated microbiomes across disparate ecosystems [9,15,17]. Consequently, this work elevates the recognized role of urban trees from passive particulate filters to active, microbiome-enhanced bioremediation systems. Such a function represents a critical and previously underappreciated ecosystem service for improving air quality in densely populated metropolitan areas [7,8,20].

To translate these mechanistic insights into practical applications, a clear roadmap for future research emerges. Priority should be placed on validating the PAH degradation functions of the key bacterial taxa identified in this study through controlled in vitro experiments [45,46,48]. Subsequently, the efficacy of designed bacterial consortia, potentially augmented with biosurfactants, should be tested on selected urban tree species under real-world conditions [49,55,58]. Ultimately, integrating these biological data into atmospheric models will be essential to predict the large-scale impact of optimized tree selections and microbiome management strategies on urban air PAH levels [20,25,30].

## 5. Conclusions

This study deciphers the drivers of phyllosphere bacterial assembly and their PAH degradation potential in Beijing’s traffic corridors. We found that bacterial community assembly is governed not by stochasticity but deterministically by leaf traits (area, morphology) and sampling height. Environmental selection was particularly strong on medium-sized (11.8–40.1 cm^2^), simple leaves positioned below 2.3 m or above 3 m. Notably, while leaf-accumulated PAHs were primarily LMW species, the overall bacterial community structure correlated significantly with HMW PAHs (e.g., benz(a)anthracene, benzo[e]pyrene, picene), underscoring a targeted role in mitigating recalcitrant traffic-derived PAHs. Furthermore, we identified a core set of degraders, including 10 dominant species (e.g., *Kocuria rosea*, *Serratia symbiotica*) from abundance correlations and 14 key species across six genera (e.g., *Acinetobacter*, *Nocardioides*) from functional predictions. These insights lead to a strategic framework for enhancing urban phytoremediation. The framework integrates the optimization of plant selection for species with medium-sized, simple leaves at mid–low heights, the construction of tailored bacterial consortia, and the application of biosurfactants to improve PAH bioavailability and degradation efficiency.

## Figures and Tables

**Table 1:** Plant species sampled, leaf traits, and sampling groups. The table lists the 20 plant species collected from Beijing’s traffic corridors, along with their mean leaf area (cm^2^), sampling height above ground (m), leaf type (SL: simple leaf; CL: compound leaf), and the assigned groups for leaf area (A1–A4) and sampling height (H1–H4).

Plant ID	Species Name	Average Leaf Area (cm^2^)	Group-Area	Sampling Height (m)	Group-Height	Group-Leaf Type	Group-PAHs Concentration	
							High	Low
P1	*Chaenomeles speciosa* (Sweet) Nakai	19.56	A2	1.5	H3	SL	P1H	P1L
P2	*Fraxinus chinensis* Roxb.	15.55	A2	4.9	H2	CL	P2H	P2L
P3	*Ailanthus altissima* (Mill.) Swingle	35.74	A3	2.2	H1	CL	P3H	P3L
P4	*Salix babylonica* L.	5.23	A4	3.2	H4	SL	P4H	P4L
P5	*Jasminum nudiflorum* Lindl.	0.75	A4	1.4	H3	CL	P5H	P5L
P6	*Populus tomentosa* Carrière	30.16	A3	5.5	H2	SL	P6H	P6L
P7	*Acer pictum* subsp. mono (Maxim.) Ohashi	23.9	A2	2.3	H1	SL	P7H	P7L
P8	*Robinia pseudoacacia* L.	7.24	A4	2	H1	CL	P8H	P8L
P9	*Prunus persica* ‘Duplex’	34.27	A3	1.5	H3	SL	P9H	P9L
P10	*Prunus triloba* Lindl.	18.47	A2	1.9	H1	SL	P10H	P10L
P11	*Broussonetia papyrifera* (L.) L ’Hér. ex Vent.	33.59	A3	3.7	H4	SL	P11H	P11L
P12	*Platanus orientalis* L.	64.11	A1	4.6	H2	SL	P12H	P12L
P13	*Ginkgo biloba* L.	18.63	A2	3.1	H4	SL	P13H	P13L
P14	*Viburnum opulus* sub sp. Calvescens (Rehder) Sugim.	40.18	A3	1.5	H3	SL	P14H	P14L
P15	*Ulmus pumila* L.	16.32	A2	4.4	H2	SL	P15H	P15L
P16	*Lonicera maackii* (Rupr.) Maxim.	11.86	A2	1.6	H3	SL	P16H	P16L
P17	*Styphnolobium japonicum* (L.) Schott	7.29	A4	3.5	H4	CL	P17H	P17L
P18	*Paulownia fortunei* (Seem.) Hemsl.	88.59	A1	4.9	H2	SL	P18H	P18L
P19	*Salix matsudana* Koidz.	12.3	A2	3	H4	SL	P19H	P19L
P20	*Prunus cerasifera* ‘Atropurpurea’	15.85	A2	1.7	H3	SL	P20H	P20L

## Data Availability

The original contributions presented in this study are included in the article/Supplementary Materials. Further inquiries can be directed to the corresponding authors.

## Supplementary Materials

The following supporting information can be downloaded at https://www.mdpi.com/article/10.3390/microorganisms14020334/s1, Text S1. PAH Analysis in leaf samples, Text S2. Data Analysis.
