# Supplementary material for: Leaf Traits Mediate Phyllosphere Bacterial Community Assembly and Their Role in Degrading Traffic-Derived Polycyclic Aromatic Hydrocarbons
Source: Microorganisms. 2026 Feb 1;14(2):334. doi: 10.3390/microorganisms14020334 (PMC12943549; doi:10.3390/microorganisms14020334)
Supplement: Supplementary file 1 [file microorganisms-14-00334-s001.zip › microorganisms-4110389-supplementary to conversion.pdf]

microorganisms

MDPI

Supplementary Materials

# Leaf Traits Mediate Phyllosphere Bacterial Community Assembly and Their Role in Degrading Traffic-Derived Polycyclic Aromatic Hydrocarbons

**Zheng Yang [1], Qingyang Liu [2,*],Shili Tian [3], Yanju Liu [3,*], Ming Yang [3], Ying Liang [3] and Xin Chen [1]**

[1] Beijing Milu Ecological Research Center, Beijing 100076, China; yz@milupark.org.cn (Z.Y.), lvrichu@126.com (X.C.)

[2] College of Ecology and the Environment, Nanjing Forestry University, Nanjing 210037, China

[3] Institute of Analysis and Testing, Beijing Center for Physical and Chemical Analysis, Beijing Academy of Science and Technology, Beijing 100089, China

* Correspondence: qyliu@njfu.edu.cn (Q.L.); liuyanju@bcpca.ac.cn (Y.L.)

S1. PAH Analysis in leaf samples

The analysis of polycyclic aromatic hydrocarbons (PAHs) on leaf surfaces followed the method described by Yang et al. (2024). Approximately 200 g of leaf sample was ground and homogenized. Subsequently, 5 g of the powder was spiked with surrogate standards (2-fluorophenyl and terphenyl-d14) and extracted ultrasonically with a mixture of n-hexane and dichloromethane (1:1, v/v). The combined extracts were dehydrated, concentrated, and purified using a solid-phase extraction column (800 mg silica gel + 1200 mg neutral alumina). The eluate was concentrated, spiked with internal standards (naphthalene-d8, acenaphthene-d10, phenanthrene-d10, chrysene-d12, and perylene-d12), and analyzed by gas chromatography–mass spectrometry (GC–MS, Shimadzu QP2010Ultra) equipped with a DB-EUPAH capillary column.

The method demonstrated good linearity ($R^2 > 0.99$) over a concentration range of 1–500 ng $mL^{-1}$. Limits of quantification for individual PAHs ranged from 0.8 to 2.8 ng $g^{-1}$, with recovery rates of 71–98%. Relative deviations for parallel samples were within 15%.

S2. Data Analysis

A suite of bioinformatic and statistical analyses were conducted. Sequencing depth was assessed using rarefaction curves. Core microbiome analysis defined operational taxonomic units (OTUs) present in at least 80% of samples within each group (Surface and Surface CK). Venn diagrams visualized overlaps between communities. The Normalized Stochasticity Ratio (NST) was calculated to quantify the relative influence of deterministic versus stochastic pro-

cesses on community assembly. Mantel tests assessed correlations between bacterial community structure (Bray–Curtis distance) and PAH concentration profiles, both overall and for subgroups defined by leaf traits (area, height, morphology). Spearman correlation analysis was used to examine relationships between (i) the relative abundance of the top 20 dominant bacterial species and PAH concentrations, and (ii) the relative abundance of taxa predicted to be involved in aromatic compound degradation and PAH concentrations. Functional prediction was performed using the FAPROTAX database (version 1.2.1), focusing on the aromatic compound degradation group. The Variance Inflation Factor (VIF) was used to screen PAH environmental factors for multicollinearity prior to correlation analyses.

Data organization was performed in Microsoft Excel. K-means clustering and Spearman correlation analyses were conducted using SPSS Statistics (Version 26.0). Bioinformatics analyses, including OTU clustering (Uparse v7.0.1090) against the Greengenes database (Release 13.5), NST analysis (NST package v3.1.10 in R v3.3.1), Mantel tests (QIIME v2020.2.0), and FAPROTAX prediction, were performed on the Majorbio Cloud Platform.

**References**

Yang M, Liu Q, Tian S, Yang Z, Yang Y, Shao P and Liu Y 2024 Occurrences of Deposited Polycyclic Aromatic Hydrocarbons in Wax of Plant Leaves Using Laser Scanning Microscopy and Gas Chromatography–Mass Spectrometry Atmosphere 15 1165
